# Haem Biosynthesis and Antioxidant Enzymes in Circulating Cells of Acute Intermittent Porphyria Patients

**DOI:** 10.1371/journal.pone.0164857

**Published:** 2016-10-27

**Authors:** Miguel D. Ferrer, Antonia Mestre-Alfaro, Magdalena Martínez-Tomé, Lucrecia Carrera-Quintanar, Xavier Capó, Antonia M. Jiménez-Monreal, Luis García-Diz, Enrique Roche, María A. Murcia, Josep A. Tur, Antoni Pons

**Affiliations:** 1 Laboratory for Physical Activity Sciences. Research Group in Community Nutrition and Oxidative Stress. Department of Basic Biology and Health Sciences. IUNICS, University of Balearic Islands, Palma, Spain; 2 Department of Nutrition and Food Science, Regional Campus of International Excellence “Campus Mare Nostrum”, University of Murcia, Murcia, Spain; 3 Biochemistry and Cell Therapy Unit, Institute of Bioengineering, University Miguel Hernandez, Elche, Spain; 4 Department of Nutrition I, University Complutense of Madrid, Madrid, Spain; 5 CIBEROBN (Fisiopatología de la Obesidad y la Nutrición CB12/03/30038) Instituto de Salud Carlos III, Madrid, Spain; Universitat de Valencia, SPAIN

## Abstract

The aims of the present study were to explore the expression pattern of haem biosynthesis enzymes in circulating cells of patients affected by two types of porphyria (acute intermittent, AIP, and variegate porphyria, VP), together with the antioxidant enzyme pattern in AIP in order to identify a possible situation of oxidative stress. Sixteen and twelve patients affected by AIP and VP, respectively, were analysed with the same numbers of healthy matched controls. Erythrocytes, neutrophils and peripheral blood mononuclear cells (PBMCs) were purified from blood, and RNA and proteins were extracted for quantitative real time PCR (qRT-PCR) and Western-blot analysis, respectively. Porhobilinogen deaminase (PBGD) and protoporphyrinogen oxidase (PPOX) gene and protein expression was analysed. Antioxidant enzyme activity and gene expression were additionally determined in blood cells, together with protein carbonyl content in plasma. PBMCs isolated from AIP patients presented low mRNA levels of PBGD when compared to controls, while PBMCs isolated from VP patients presented a decrease in PPOX mRNA. PPOX protein content was higher in AIP patients and lower in VP patients, compared to healthy controls. Regarding antioxidant enzymes, PBMCs and erythrocyte superoxide dismutase (SOD) presented statistically significant higher activity in AIP patients compared to controls, while catalase activity tended to be lower in these patients. No differences were observed regarding antioxidant gene expression in white blood cells. Circulating cells in AIP and VP patients present altered expression of haem biosynthetic enzymes, which could be useful for the differential diagnosis of these two types of porphyria in certain difficult cases. AIP patients present a condition of potential oxidative stress similar to VP patients, evidenced by the post-transcriptional activation of SOD and possible catalase impairment.

## Introduction

Porphyrias are a group of metabolic disorders where haem biosynthesis is affected, thus resulting in the accumulation of porphyrins or their precursors in different tissues [1,2]. Although porphyrias are inherited diseases, there are certain agents that can induce acute attacks as well as the appearance of clinical symptoms [3]. Acute intermittent porphyria (AIP) is an autosomal dominant type of hepatic porphyria caused by mutations in the porphobilinogen deaminase (PBGD) gene. These mutations result in catalytic deficiency of PBGD, the third enzyme in haem biosynthesis [4], with a residual enzymatic activity approximately half the normal levels. Variegate porphyria (VP), another autosomal dominant type of hepatic porphyria, is the result of decreased protoporphyrinogen oxidase (PPOX) activity, the next-to-last enzyme in haem biosynthesis, and is characterized by skin lesions and acute attacks [2]. Chronic accumulation of haem precursors in erythrocytes, liver or other tissues is responsible for the major clinical and pathological manifestations of these diseases [1,2,5]. However, little is known regarding the gene and protein expression of PBGD and PPOX in extra-hepatic tissues.

It has been well described that the chronic accumulation of haem precursors could enhance reactive oxygen species (ROS) production and generate an unbalanced oxidative situation, especially in patients with low exogenous antioxidant defences such as porphyria patients [6–9]. In fact, erythrocytes of VP patients accumulate oxidative damage markers [10] and their lymphocytes are more susceptible to suffer from DNA damage after stress stimulation with hydrogen peroxide [11]. In addition, oxidative damage could be enhanced by low levels of endogenous and exogenous antioxidants, such as vitamin E [7,8]. In this context, a recent study has demonstrated that AIP patients present a deficient intake of antioxidant vitamins [9].

The aim of this study was to compare the expression levels of PBGD and PPOX in circulating blood cells of AIP and VP patients, and, in the case of AIP patients, to analyse the antioxidant enzyme activity in erythrocytes, peripheral blood mononuclear cells (PBMCs) and neutrophils, compared to matched controls. As for the samples taken from VP patients, these were compared with previously published data.

## Materials and Methods

### Subjects

A total of 16 AIP patients (11 females and 5 males) from Murcia (Spain) recruited through Health Centres of endemic areas were contacted and agreed to participate in the study. The AIP condition had been clinically diagnosed to all the patients included in the study. AIP patients carried a mutation in the 12 exon (30-bp deletion) 669–698 position, 11 g 23Æ3 locus, and presented PBGD enzyme activity decreased by 50%. A complete clinical, biochemical and anthropometric characterisation of these patients has already been published [9,12]. Control group was formed by 16 healthy volunteers (11 females and 5 males) who shared similar age, height and weight values as the patients.

A total of 12 women affected by VP, as well as 12 pair-matched healthy control women, were also recruited for the study using the Balearic Association of Porphyria, which registers all the porphyric patients in Balearic Islands (Spain). The subjects included in the VP group had been previously diagnosed by specialized doctors on the basis of different parameters such as plasma fluorescence peak at 626 nm, levels of excreted urinary and faecal porphyrins and clinical manifestations such as abdominal pain during attacks [10]. Control women were also recruited through the Balearic Association of Porphyria and were pair-matched in age with the women included in the porphyric group.

This study was conducted according to guidelines written in the Declaration of Helsinki and all procedures involving human subjects/patients were approved by the Ethical Committee of Clinical Investigation of the Balearic Islands and the Ethics Committee of Clinical Investigation of the University of Murcia. Written informed consent was obtained from all subjects.

Venous blood samples were obtained from the antecubital vein of control and porphyric patients in resting conditions after overnight fasting and placed in EDTA vacutainers. PBMCs, neutrophils and erythrocytes were purified following an adaptation of the method described by Ferrer [13] using Ficoll-Paque PLUS reagent (GE Healthcare, Little Chalfont, United Kingdom). Briefly, blood was carefully introduced in a Corning tube on Ficoll in a proportion of 1.5:1 v:v, and centrifuged at 900xg for 30 min at 4°C. The precipitate was incubated at 4°C with 0.15 M ammonium chloride to lyse the erythrocytes. The suspension was centrifuged at 750xg, at 4°C for 15 min and supernatant was discarded. The bottom phase containing neutrophils was washed first with ammonium chloride and then with phosphate-buffered saline solution at pH 7.4. The intermediate PBMCs layer obtained from the Ficoll gradient was carefully removed, washed twice with PBS and centrifuged at 1000xg for 10 min at 4°C. This procedure ensures a PBMCs purity and viability of 95 ± 5%. Erythrocytes were obtained from another blood sample after 1000xg centrifugation for 15 min at 4°C. The plasma, which corresponded to the supernatant after centrifugation, was stored at -80°C. Erythrocytes present in the pellet were washed with PBS, resuspended, incubated in ice, centrifuged under the same conditions and resuspended in the same volume as the original plasma.

### mRNA gene expression

mRNA expressions were determined by quantitative real time PCR (qRT-PCR). For this purpose, mRNA was isolated from PBMCs using Tripure Isolation Reagent (Roche Diagnostics, Basel, Switzerland). cDNA was synthesized from 1 μg total RNA using reverse transcriptase with oligo-dT primers. q-RT-PCR was performed using the LightCycler instrument (Roche Diagnostics, Basel, Switzerland) with DNA-master SYBR Green I (Roche Diagnostics, Basel, Switzerland). All PCRs were performed with the same basic program, with one cycle at 95°C for 10 min, followed by 40 cycles at the conditions shown in Table 1. The primers used for amplification are also shown in Table 1.

**Table 1:** Primer sequences and conditions used in the quantitative real time PCR analysis.

Gene	Primer	Conditions	
**18S**	**Fw:** 5’-ATGTGAAGTCACTGTGCCAG-3’	95°C	10 s
	**Rv:** 5’-GTGTAATCCGTCTCCACAGA-3’	60°C	7 s
		72°C	12 s
**PBGD**	**Fw:** 5’-cacagcactcccactgacaa-3’	95°C	10 s
	**Rv:** 5’-gagtggggaaatactccaagg-3’	58°C	10 s
		72°C	15 s
**PPOX**	**Fw:** 5’-TGCCGCGAGAACAGAGTGGAC-3’	95°C	10 s
	**Rv:** 5’-GGCCCATGCGGAAACCCACA-3’	60°C	10 s
		72°C	15 s
**CAT**	**Fw:** 5’-TTTGGCTACTTTGAGGTCAC-3’	95°C	10 s
	**Rv:** 5’-TCCCCATTTGCATTAACCAG-3’	60°C	10 s
		72°C	12 s
**Mn-SOD**	**Fw:** 5’-CGTGCTCCCACACATCAATC -3’	95°C	10 s
	**Rv:** 5’-TGAACGTCACCGAGGAGAAG-3’	60°C	10 s
		72°C	15 s
**GPX**	**Fw:** 5'-TTCCCGTGCAACCAGTTTG-3'	95°C	10 s
	**Rv:** 5'-TTCACCTCGCACTTCTCGAA-3'	63°C	10 s
		72°C	15 s
**Cu/Zn-SOD**	**Fw:** 5’-TCAGGAGACCATTGCATCATT-3’	94°C	10 s
	**Rv:** 5’-CGCTTTCCTGTCTTTGTACTTTCTTC-3’	63°C	10 s
		72°C	15 s
**MPO**	**Fw:** 5’-TGAACATGGGGAGTGTTTCA-3’	95°C	15s
	**Rv:** 5’-CCAGCTCTGCTAACCAGGAC-3’	61°C	30s
		72°C	60 s

Abbreviations used: 18S: Ribosomal RNA 18S. CAT: Catalase. GPX: Glutathione peroxidase. MPO: Myeloperoxidase. PBGD: Porphobilinogen deaminase. PPOX: Protoporphyrinogen oxidase. SOD: Superoxide dismutase.

The relative quantification was performed by standard calculations considering 2^(-ΔΔCt)^. Basal mRNA levels at the beginning of the stage were arbitrarily referred to as 1. The expression of the target gene was normalized with respect to ribosomal 18S.

### Western blot analysis

PPOX and PBGD protein levels were determined by Western blot. PBMCs samples were treated with RIPA (radioimmunoprotection assay) buffer and total protein concentrations were measured by the method of Bradford [14]. Protein extracts were analysed by SDS-polyacrylamide gel electrophoresis (SDS-PAGE). For PPOX, 75 μg of total protein were loaded on a 12.5% agarose gel. Following electrophoresis, samples were transferred onto a nitrocellulose membrane and incubated with a primary monoclonal anti-PPOX antibody (Santa Cruz Biotech, Heidelberg, Germany) and a secondary anti-goat IgG peroxidase-conjugated antibody. The same procedure was performed for PBGD, only 30 μg of total protein was used and incubated using a primary monoclonal anti-PBGD antibody (Santa Cruz Biotech, Heidelberg, Germany) and a secondary anti-goat IgG peroxidase-conjugated antibody. In both cases the signal was visualized using luminol, which is converted into a light-emitting form at wavelength 428 nm by the antigen/primary, antibody/secondary, antibody/peroxidase complex. The light was visualized and detected by short exposure to a Molecular Imager Chemidoc XRS (Bio-Rad Laboratories, Hercules, CA, USA). Image analysis was performed using Quantity One-1D analysis software (Bio-Rad Laboratories, Hercules, CA, USA).

### Protein carbonyl determination

Protein carbonyl derivatives were determined in plasma by immunological methods using the OxiSelectTM Protein Carbonyl Immunoblot Kit (Cell Biolabs, INC) by following the manufacturer’s details. Total plasma protein concentrations were measured by the Bradford method [14] using the Bio-Rad protein assay reagent (Bio-Rad Laboratories, Hercules, CA, USA). Initially, 10 μg of protein were transferred onto a nitrocellulose membrane by the dot blot method. The membrane was incubated in the presence of2,4-dinitrophenylhydrazine (DNPH) after transference. Then, the membrane was incubated with the primary antibody, specific to DNP moiety proteins (1:4,000). This step was followed by incubation with a horseradish peroxidase-antibody (goat anti-rabbit IgG) conjugate directed against the primary antibody (1:10,000). The membrane was then treated with luminol, which is converted to a light-emitting form at wavelength 428 nm by the antigen/primary antibody/secondary antibody/peroxidase complex. The light was visualized and detected by short exposure to a Chemidoc XRS densitometer (Bio-Rad Laboratories, Hercules, CA, USA). Image analysis was performed using Quantity One-1D analysis software (Bio-Rad Laboratories, Hercules, CA, USA).

### Antioxidant enzyme activities

Superoxide dismutase (SOD), catalase and glutathione peroxidase (GPX) activities were measured in PBMCs, neutrophils and erythrocytes as previously published [10,13,15,16]. Myeloperoxidase (MPO) activity was determined in neutrophils by the guaiacol oxidation method [17].

### Statistical analysis

Statistical analysis was performed using the statistical package for social sciences (SPSS 19.0 for Windows). Results are expressed as mean ± SEM (standard error of the mean) and p<0.05 was considered statistically significant. The statistical significance was assessed by Student’s t-test for unpaired data.

## Results

**Fig 1:**
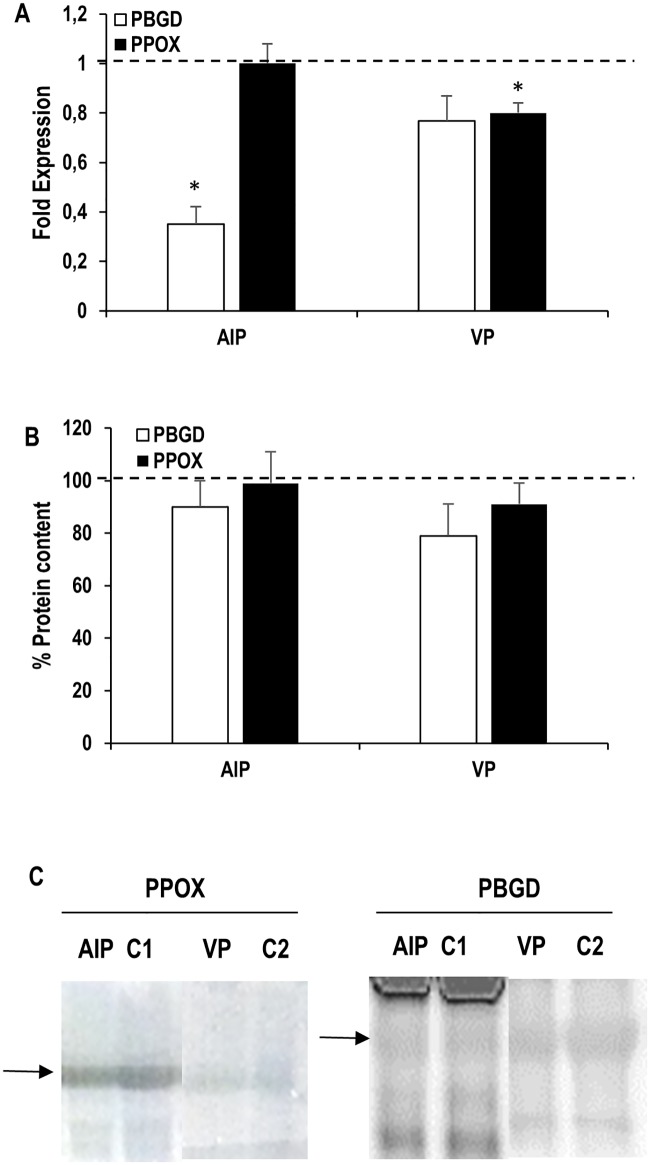
Effects of acute intermittent porphyria (AIP) and variegate porphyria (VP) on PBMCs porphobilinogen deaminase (PBGD) and protoporphyrinogen oxidase (PPOX) gene expression (A) and protein content (B, C). (A) The relative quantification was performed by standard calculations considering 2^(-ΔΔCt)^. mRNA levels of control subjects were arbitrarily referred to as 1 (dashed line). (B) Total protein content in the control group was normalized to 100% (dashed line). Results are expressed as mean ± sem. Statistical analysis: Student’s t-test for unpaired data. (*) indicates statistically significant differences between porphyria and control groups (p < 0.05). C1: Control for AIP patients. C2: Control for VP patients.

**Fig 2:**
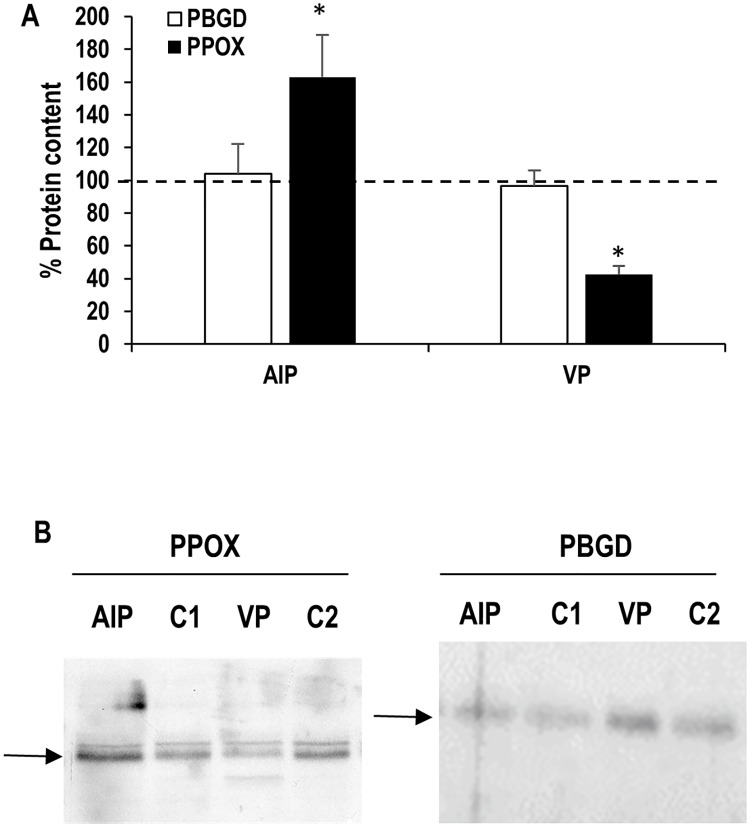
Effects of acute intermittent porphyria (AIP) and variegate porphyria (VP) on erythrocyte porphobilinogen deaminase (PBGD) and protoporphyrinogen oxidase (PPOX) protein content. Total protein content in the control group was normalized to 100% (dashed line). Statistical analysis: Student’s t-test for unpaired data. (*) indicates statistically significant differences between porphyria and control groups (p < 0.05). C1: Control for AIP patients. C2: Control for VP patients.

Gene expression and protein content of both PBGD and PPOX enzymes were analysed in PBMCs from AIP and VP patients and their respective controls in order to verify alterations in the haem biosynthetic pathway (Fig 1). AIP patients presented statistically significant lower PBGD gene expression (approximately 35%) than their controls, while PPOX expression did not differ between groups (Fig 1A). This difference, however, was not observed at the protein level (Fig 1B). On the other hand, erythrocyte PBGD protein levels did not differ between AIP patients and controls, while PPOX protein levels were 60% higher in AIP patients compared to healthy control individuals (Fig 2A).

On the other hand, PBMCs isolated from VP patients presented decreased PPOX gene expression (about 75% of control) and similar PBGD expression compared to control subjects (Fig 1A). Again, the differences observed in gene expression were not detected at the protein level, similar to what was observed in AIP patients (Fig 1B). VP patients presented lower erythrocyte PPOX protein levels compared to healthy controls (Fig 2A), with no differences detected in PBGD protein levels.

Although PBGD and PPOX protein levels were similar to controls in the PBMCs of both types of porphyria, it is very likely that the enzymatic activities are altered, leading to accumulation of haem biosynthesis intermediates and causing oxidative stress and defective function of haem-containing enzymes, such as catalase. For that reason, antioxidant enzyme activities were analysed in PBMCs, erythrocytes and neutrophils of AIP patients and compared to control subjects (Table 2) and to previously published data in VP patients [10,15,16].

**Table 2:** Effect of acute intermittent porphyria (AIP) on blood cell antioxidant enzyme activities and plasma protein carbonyl content.

	Control (N = 16)	AIP (N = 16)	P
**PBMCs**			
**Catalase (K/10**^**9**^ **cell)**	8.97 ± 2.23	4.47 ± 0.81	0.068
**SOD (pkat/10**^**9**^ **cell)**	3.42 ± 0.16	4.30 ± 0.31*	< 0.05
**GPX (nkat/10**^**9**^ **cell)**	103 ± 10	133 ± 22	> 0.1
**Erythrocytes**			
**Catalase (K/10**^**9**^ **cell)**	3.14 ± 0.30	3.99 ± 0.74	> 0.1
**SOD (nkat/10**^**6**^ **cell)**	4.09 ± 0.11	4.38 ± 0.10 *	< 0.05
**GPX (nkat/10**^**6**^ **cell)**	428 ± 53	358± 52	> 0.1
**Neutrophils**			
**Catalase (K/10**^**9**^ **cell)**	52.6 ± 4.6	39.8 ± 7.0	> 0.1
**SOD (pkat/10**^**9**^ **cell)**	0.98 ± 0.07	1.12 ± 0.09	> 0.1
**GPX (nkat/10**^**9**^ **cell)**	22.6 ± 3.2	29.9 ± 5.5	> 0.1
**MPO (nkat/10**^**9**^ **cell)**	6.76 ± 1.62	14.1 ± 3.5	0.066
**Plasma**			
**Protein carbonyl derivatives (%)**	100 ± 23	88.1 ± 8.9	> 0.1

Abbreviations used: PBMCs: Peripheral blood mononuclear cells. GPX: Glutathione peroxidase. MPO: Myeloperoxidase. SOD: Superoxide dismutase. Enzymatic activities are referred to katals (mol of substrate transformed/s), except for catalase which is referred to K (rate constant of the first order reaction). Results are expressed as mean ± sem. Statistical analysis: Student’s t-test for unpaired data. (*) indicates statistically significant differences between porphyria and control groups (p < 0.05).

PBMCs SOD activity was significantly higher in AIP patients than in controls Catalase activity was approximately half normal levels in patients, although this difference was not statistically significant (p = 0.063). GPX activity was slightly, but not significantly, higher in AIP patients. Neutrophils presented an antioxidant enzyme activity pattern similar to that observed in PBMCs, although the differences between groups were not statistically significant (Table 2). In addition, activity of the pro-oxidant/inflammatory enzyme MPO was 2-fold higher in neutrophils from AIP patients, compared to controls (p = 0.066). Erythrocytes only presented increased SOD activity, while catalase and GPX were unaffected. Protein carbonyl content was additionally measured in plasma as a marker of systemic oxidative damage. As seen in Table 2, no differences in protein carbonyl content were observed between AIP patients and controls.

Antioxidant gene expression was additionally assessed in white blood cells in order to explain the possible mechanisms behind the antioxidant response observed in enzyme activity. Antioxidant gene expression did not differ between AIP patients and controls (Table 3). Neutrophil MPO gene expression was slightly elevated in AIP patients when compared to controls, but this difference was not statistically significant.

**Table 3:** Effect of acute intermittent porphyria (AIP) on blood cell antioxidant enzyme gene expression.

	Control (N = 16)	AIP (N = 16)	P
**PBMCs**			
**Catalase**	1.00 ± 0.16	1.11 ± 0.19	> 0.1
**Mn-SOD**	1.00 ± 0.22	1.07 ± 0.24	> 0.1
**Cu/Zn-SOD**	1.00 ± 0.16	0.93± 0.13	> 0.1
**GPX**	1.00 ± 0.19	0.87 ± 0.11	> 0.1
**Neutrophils**			
**Catalase**	1.00 ± 0.25	1.20 ± 0.26	> 0.1
**Mn-SOD**	1.00 ± 0.36	1.06 ± 0.35	> 0.1
**Cu/Zn-SOD**	1.00 ± 0.24	0.95 ± 0.27	> 0.1
**GPX**	1.00 ± 0.29	0.99 ± 0.18	> 0.1
**MPO**	1.00 ± 0.25	2.80 ± 1.32	> 0.1

Abbreviations used: AIP: Acute intermittent porphyria. GPX: Glutathione peroxidase. MPO: Myeloperoxidase. PBMCs: Peripheral blood mononuclear cell. SOD: Superoxide dismutase. The relative quantification was performed by standard calculations considering 2^(-ΔΔCt)^. mRNA levels of control subjects were arbitrarily referred to as 1. Results are expressed as mean ± sem. Statistical analysis: Student’s t-test for unpaired data. No statistically significant differences between porphyria and control groups were detected (p < 0.05).

### Discussion

The present study indicates that PBMCs isolated from AIP and VP patients possess different expression patterns of the enzymes that cause the disease. Specifically, while in AIP patients there are lower PBGD gene expression levels in PBMCs, VP patients present lowered PPOX gene expression. Although more than 130 mutations in the PBGD gene have been identified in relation to AIP, all patients manifest the same symptoms and the residual PBGD enzyme activity is approximately half normal levels, independent of the mutation carried. The present study is the first to demonstrate that AIP patients have lower PBGD gene expression in PBMCs compared to healthy individuals. This decrease in expression is not attributable to a technical artefact of the qRT-PCR technique, because the primers used in this study were not targeted to the mutated sequence, which could have impaired a correct annealing and resulted in a lower number of amplicons. Therefore, the most likely explanation is that the AIP patients analysed in this study actually presented lower PBGD mRNA levels in PBMCs than their respective controls. In accordance with our observation, a previous study reported that PBGD mRNA levels were found to be less than half in brains of Alzheimer’s disease patients than in controls [18]. The lower levels observed could be attributed to a lower degree of gene expression or to a decreased RNA stability, or both. Regarding the first possibility, it is worth mentioning that PBGD mRNA levels are not regulated by haem levels in mammalian cells. Control by haem levels is exerted in the first enzyme of the pathway, the aminolevulinic acid synthase, which increases its activity as a result of the low levels of haem due to the deficiency of PBGD in AIP [19]. This, in turn, gives rise to a deregulation of the pathway, since PBGD is a known regulator of haem synthesis. Therefore, the determinants that modulate PBGD gene expression in PBMCs from AIP patients remain to be identified, although the possibility of increased PBGD mRNA instability also needs to be explored.

A similar rationale can be assessed for the PPOX gene in PBMCs from VP patients, although the decrease in expression seems to be less prominent than in the case of the PBGD gene for AIP patients (only 20%). Furthermore, the determination of PBGD and PPOX protein levels in PBMCs does not present the differences observed using mRNA (Fig 2). The explanation for this observation in PBMCs seems to be related to changes in protein turnover involving faster protein synthesis or reduced protein degradation in porphyria patients in order to compensate for the lower RNA levels, although further studies are necessary to verify this point. However, PBGD and PPOX protein levels follow a different behaviour in erythrocytes, pointing out the differences between this cell type and PBMCs. In erythrocytes from AIP patients, PPOX content is increased (as indicated by its higher protein levels) when compared to healthy controls. Although PBGD erythrocyte protein content is not decreased in AIP patients, the resulting enzyme is less efficient and its enzyme activity is reduced to almost half of control activity [19]. Our results of PPOX protein expression point out that maturating erythrocytes (a cell type with a high demand of haem groups) respond to the decrease in PBGD activity (located in the first reactions of the biosynthesis pathway) with increased levels of the PPOX enzyme (next-to-last enzyme in the haem synthesis pathway) in order to partially compensate PBGD reduced activity to maintain haem levels. The specifically different pattern of PPOX protein expression in the erythrocytes of AIP and VP patients (overexpressed in AIP patients and underexpressed in VP patients) suggests that this laboratory determination could be useful in cases of patients whose mutation has not been identified and the symptomatology does not allow to differentiate AIP from VP. However, a proper technical and clinical validation should be performed previous to the application of this determination on the clinical practice.

In any case, the impaired enzyme activities lead to the accumulation of porphyrin intermediates that can be easily oxidized and thus result in increased oxidative stress in affected tissues [20,21]. The accumulation of α-aminolevulinic acid (α-ALA) [22] and other porphyrins [23] can induce oxidative damage through a direct action or through the production of reactive oxygen species, thus inducing a situation of oxidative stress. Together with these direct effects of the intermediate compounds on ROS production or oxidative damage, the porphyric condition can also induce oxidative stress through other ways. The haem group is an essential component of several proteins; therefore, the limitations in the synthesis of this prosthetic group can imply a limited function of these proteins. In the first instance, impaired haem synthesis can affect the functionality of the components in the mitochondrial respiratory chain such as cytochromes, thus inducing higher ROS release. In fact, it has been previously reported that the lymphocytes from women affected by VP produce greater amounts of H_2_O_2_ by the complex III of the mitochondrial respiratory chain than control women after ROS production stimulation [11].

In addition to the higher ROS production presented by porphyria patients, the accumulation of porphyrins could also affect the functionality of antioxidant enzymes such as catalase, thus impairing the ROS detoxifying capacity. Catalase is a major antioxidant enzyme that presents a tetrameric structure containing one haem group in each subunit. This enzyme detoxifies the H_2_O_2_ produced by SOD during the reduction of the superoxide anion. In this context, and compared to controls, catalase activity tends to present a slightly lower activity in AIP patients. These results are in accordance with those previously reported in VP patients, who presented lower catalase activity in PBMCs and neutrophils [15,16], evidencing that deficiency in haem biosynthesis negatively affect catalase activity.

Regarding the remaining antioxidant enzymes in circulating blood cells, SOD also seems to present a similar pattern of activity in AIP and VP patients, mainly in erythrocytes. AIP patients present higher SOD activity in circulating cells (no statistically significant for neutrophils) when compared to controls, and erythrocyte SOD activity in VP patients is also higher than in their respective controls [10]. Concerning the possible mechanisms of regulation involved in this increased SOD activity, and although it could be related to increased enzyme synthesis in erythrocyte cell precursors, in previous studies we have evidenced that the presence of oxidative stress can directly enhance the activity of antioxidant enzymes such as glutathione reductase and superoxide dismutase in the mature erythrocyte through post-transcriptional mechanisms [24,25]. The results obtained with the analysis of SOD gene expression in PBMCs are in accordance with this post-transcriptional mechanism of activation of the enzyme, as PBMCs present higher SOD activity while the expression of both Mn- and Cu/Zn-SOD is equivalent between AIP patients and controls. Therefore, the higher erythrocyte SOD activity found in women affected by AIP and VP could be a consequence of enzyme activation due to the exposure of these cells to increased ROS levels produced by the accumulated porphyrins (erythrocytes) or the respiratory chain (PBMCs).

Although a precise picture of the antioxidant pattern observed in porphyria patients is complicated, our results allow us to hypothesize on a first model that might explain this antioxidant response. SOD activity is the first line of defence against ROS and is activated by the presence of increased levels of ROS, transforming superoxide anion into H_2_O_2_, an important cytotoxic intermediate. Hypothetically, AIP patients should produce higher amounts of H_2_O_2_ since they present higher SOD activity compared to controls. However, and independently from the H_2_O_2_ levels produced in circulating blood cells, this toxic compound cannot be properly detoxified by catalase activity, a haem-containing enzyme which tends to be defective in porphyria patients. Therefore, GPX could be postulated as the main pathway to eliminate H_2_O_2_. Although GPX activity in porphyria patients seems to be normal, glutathione is necessary for this enzyme to function properly. In this context, intracellular levels of glutathione are dependent of many environmental factors which include diet antioxidants that in many cases are deficient in AIP patients, such is the case for vitamin E [9]. Taking all these results altogether (and those previously reported for VP), the circulating cells of porphyria patients (both AIP and VP) generate a higher amount of H_2_O_2_ than healthy controls with a potential impairment in the detoxifying mechanism for these compound, facing these cells with a potential situation of oxidative stress which is apparently not manifested in basal, non-prompted conditions, as evidenced by the similar protein carbonyl content between AIP patients and controls. This picture is very similar to that previously described in VP patients, who also do not present protein or DNA oxidative damage in basal conditions but are more susceptible to develop oxidative damage in situations of increased ROS production [11,15]. Therefore, taking into account the results obtained with the aforementioned nutritional status study and the new results reported in this study, the diet of porphyria patients should be specifically controlled by the doctor and should be rich in olive oil, vegetables and fruits with high contents of antioxidants in order to reinforce the activities of glutathione-dependent enzymes and the overall antioxidant status of their organism. In fact, the diet supplementation of VP patients with an almond beverage enriched with vitamins C and E was shown to stimulate the endogenous antioxidant enzyme defences and to reduce oxidative damage and even restored PPOX gene expression in lymphocytes [15,16], pointing out the importance of the antioxidant intake in the maintenance of the oxidant/antioxidant status of the organism, especially in these predisposed subjects.

In conclusion, patients affected by AIP present reduced mRNA expression of PBGD and normal expression of PPOX in circulating PBMCs, while patients affected by VP present with the exact opposite picture. The determination of PPOX protein levels in erythrocytes could be a potential diagnostic tool to discriminate AIP from VP in difficult cases in which the gene sequencing and other diagnostic factors are not conclusive. Patients affected by AIP present a situation of potential oxidative stress similar to those affected by VP and characterized by increased H_2_O_2_ production by post-transcriptionally activated SOD and impaired H_2_O_2_ detoxifying mechanisms, such as catalase, pointing out that the supplementation of the diet of these patients with higher intakes of antioxidants could ameliorate its pathological condition.
